# Ablation of VLA4 in multiple myeloma cells redirects tumor spread and prolongs survival

**DOI:** 10.1038/s41598-021-03748-0

**Published:** 2022-01-07

**Authors:** Deep Hathi, Chantiya Chanswangphuwana, Nicholas Cho, Francesca Fontana, Dolonchampa Maji, Julie Ritchey, Julie O’Neal, Anchal Ghai, Kathleen Duncan, Walter J. Akers, Mark Fiala, Ravi Vij, John F. DiPersio, Michael Rettig, Monica Shokeen

**Affiliations:** 1 Department of Biomedical Engineering, Washington University in St. Louis, St. Louis, MO USA; 2 Department of Medicine, Division of Molecular Oncology, Washington University School of Medicine, St. Louis, MO USA; 3 Department of Medicine, Division of Hematology, Chulalongkorn University and King Chulalongkorn Memorial Hospital, Bangkok, Thailand; 4 Department of Medicine, Division of Cardiology, Washington University School of Medicine, St. Louis, MO USA; 5 Department of Radiology, Washington University School of Medicine, St. Louis, MO USA; 6 Center for In Vivo Imaging and Therapeutics, St. Jude Children’s Research Hospital, Memphis, TN USA

**Keywords:** Cancer, Cancer imaging, Haematological cancer

## Abstract

Multiple myeloma (MM) is a cancer of bone marrow (BM) plasma cells, which is increasingly treatable but still incurable. In 90% of MM patients, severe osteolysis results from pathological interactions between MM cells and the bone microenvironment. Delineating specific molecules and pathways for their role in cancer supportive interactions in the BM is vital for developing new therapies. Very Late Antigen 4 (VLA4, integrin *α*_4_*β*_1_) is a key player in cell–cell adhesion and signaling between MM and BM cells. We evaluated a VLA4 selective near infrared fluorescent probe, LLP2A-Cy5, for in vitro and in vivo optical imaging of VLA4. Furthermore, two VLA4-null murine 5TGM1 MM cell (KO) clones were generated by CRISPR/Cas9 knockout of the *Itga4* (*α*_4_) subunit, which induced significant alterations in the transcriptome. In contrast to the VLA4^+^ 5TGM1 parental cells, C57Bl/KaLwRij immunocompetent syngeneic mice inoculated with the VLA4-null clones showed prolonged survival, reduced medullary disease, and increased extramedullary disease burden. The KO tumor foci showed significantly reduced uptake of LLP2A-Cy5, confirming in vivo specificity of this imaging agent. This work provides new insights into the pathogenic role of VLA4 in MM, and evaluates an optical tool to measure its expression in preclinical models.

## Introduction

Multiple myeloma (MM) is a debilitating cancer of bone marrow (BM) plasma cells, causing damage to multiple organs including bone and kidneys. The 5-year overall survival in MM patients has improved from 26.5% in 1975 to 56.85% in 2013^1^. The ~ twofold improvement in relative survival over the last four decades has largely resulted from the use of high-dose chemotherapy with stem cell transplant, proteasome inhibitors, and monoclonal antibody therapies^2^. The grim reality, however, is the frequent relapse of refractory disease in MM patients. There remains an urgent need to identify underlying mechanisms that drive myeloma cells from a treatment responsive state to a treatment resistant state.

In addition to the cell autonomous factors such as high-risk cytogenetics, the bone marrow (BM) microenvironment is co-opted by myeloma cells for homing, survival, and acquiring drug resistance. The dynamic of interactions between myeloma cells and the microenvironmental factors fosters their ability to grow, home to new sites, escape immune surveillance and resist chemotherapy. The clinical impact of MM on bone health is apparent from the fact that over 80% of patients present with osteolytic lesions at diagnosis, with increased risk of skeletal related events^3–5^. About 60% of MM patients will suffer a fracture during the disease course despite treatment, with the severity of bone lesions serving as an independent predictor of survival in MM patients^6^. In advanced stages, 3–6% of patients will likely present with extramedullary myeloma, an aggressive sub-type of MM, in which the myeloma tumors are able to grow independent of BM support^7^.

The disruption of the vicious feedback loop between MM cells and bone microenvironment could potentially prevent osteolytic disease and sensitize MM cells to various therapies. While multiple factors play a role in myeloma carcinogenesis, the Very Late Antigen 4 (VLA4, CD49d/CD29, integrin α_4_β_1_) is a front-runner implicated in myeloma cell homing, survival and acquisition of cell-adhesion mediated drug resistance (CAM-DR). VLA4 is a noncovalent, heterodimeric transmembrane receptor that is over-expressed on MM cells and its natural ligands are vascular cell adhesion molecule-1 (VCAM-1) and fibronectin^8^. VLA4 exists in equilibrium between different conformational states and its binding is dynamically regulated with bidirectional (inside out and outside in) signaling^9^. The conformational changes in VLA4 from low-affinity state to high-affinity/activated state is reflective of functional VLA4, which mediates intracellular signaling pathways such as integrin-linked kinases, focal adhesion kinases and Src-family kinases^10^. The activated form of VLA4 binds with high affinity to the ligands, fibronectin and VCAM-1 expressed on BM stromal cells, increasing myeloma cell adhesion and survival within the BM microenvironment^11,12^. The interaction between VLA4 on myeloma cells and VCAM-1 in the bone microenvironment reduces osteoblastogenesis, increases osteoclastogenesis and promotes myeloma bone disease^13–17^.

Additionally, an enhanced level of VLA4 on MM cells indicates a drug-resistant phenotype^18^ and is implicated in CAM-DR^16^. In fact, the protective effect provided by stromal cell contact can be reversed with anti-α_4_ antibodies or α_4_-knock down genetic approaches^19–21^**.** Furthermore, clinical and preclinical data show that VLA4 is overexpressed in samples of minimal residual disease and drug-refractory MM cells. Shain et al.showed that drug resistance of myeloma cell lines correlates with increased adherence potential (i.e., CAM-DR), via either increased VLA4 expression or shift from low- to high- affinity state^16,22,23^. Leveraging on this phenomenon, preclinical data show that upregulation of VLA4 can enhance effective delivery of VLA4-targeted drugs to drug-resistant MM cells in mice^24^.

Characterization and monitoring of activated VLA4 conformation in different organs and during various phases of MM development using advanced imaging methods can be a powerful tool for evaluating myeloma cell’s interaction with BM cellular and extra-cellular components. We hypothesized that the downregulation of VLA4 in MM cells will confer a survival advantage due to the impaired ability of these cells to adhere to the BM stroma. Furthermore, this process can be seamlessly interrogated in vitro and in vivo using molecularly targeted imaging agents. This work presents (1) the development and characterization of both an immunocompetent isogenic model of mouse myeloma that ablates VLA4 function through CRISPR KO of its alpha subunit, *Itga4*, and (2) a novel tool to image activated VLA4 pre-clinically, *ex-vivo*, and in cells using a molecularly targeted, non-ionizing, near-infrared (NIR) imaging agent.

NIR imaging is attractive because of reduced absorption of light by melanin and other chromophores in the NIR window (650–900 nm)^25^, allowing economical and high-throughput preclinical imaging. Without the use of ionizing radiation, the same probe can be used repeatedly in vivo for whole-body imaging pre-clinically, and for following agent binding at the cellular level, allowing a precise characterization of tissues of interest. LLP2A is a selective peptidomimetic ligand with high-affinity for the activated form of VLA4. Radioactively labelled LLP2A has been validated as a nuclear imaging agent in preclinical models of MM and is currently being evaluated for safety and dosimetry in a Phase 1 clinical trial^26–28^.

In this study, we generated CRISPR/Cas9-mediated knockout (KO) of the *Itga4* (α_4_) subunit in the VLA4^+^ 5TGM1-GFP parental murine myeloma cell line (WT 5TGM1) to investigate the biological variation in vitro and in vivo resulting from VLA4 ablation. We show that VLA4 ablation in the 5TGM1 cells induced significant differences in the transcriptome, in vivo tumor dissemination, and survival in the immunocompetent 5TGM1/KaLwRij myeloma mouse model. We evaluated the VLA4 targeted NIR peptidomimetic, LLP2A-Cy5^28^, in immunocompetent mice bearing intravenous syngeneic mouse tumors (murine 5TGM1 C57Bl/KaLwRij (KaLwRij)^29^). Our results demonstrate selectivity of LLP2A-Cy5 for VLA4 in vitro and in vivo in intramedullary myeloma lesions in the tumor bearing mice. Collectively, these insights seek to inform on the off-target side effects of VLA4 modulation on MM cells and guide the design of novel VLA4 targeted interventions to safely dislodge adherent myeloma cells from the BM and potentially sensitize the cells to potent therapies.

## Results

### ***Itga4*** (α_4_) KO (VLA4-null) 5TGM1-GFP murine myeloma cells have altered integrin related signaling pathways compared to the WT 5TGM1-GFP cells

**Figure 1:**
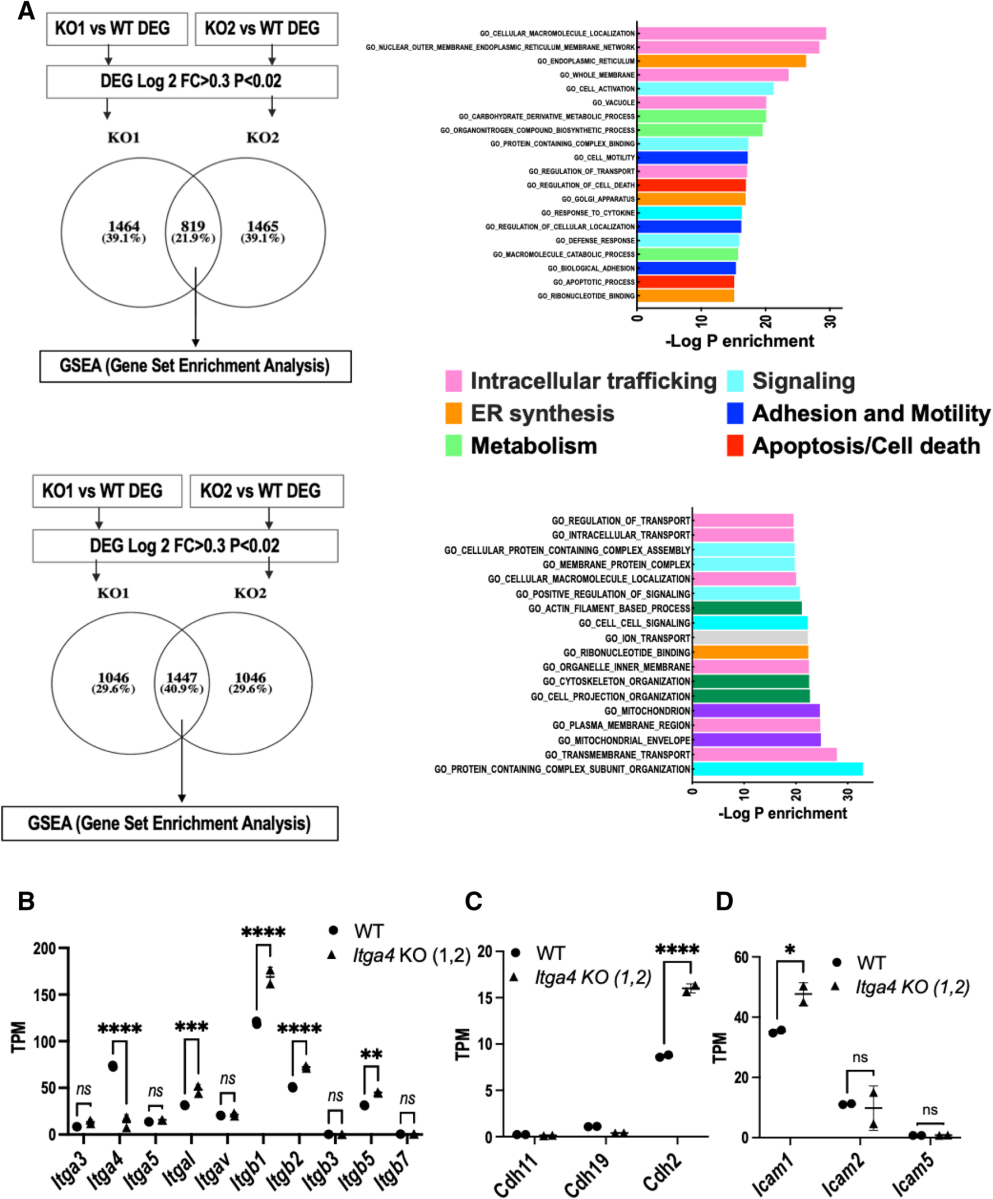
Gene set enrichment analysis (GSEA) of differentially expressed genes (DEG) by RNAseq of WT vs Itga4 KO 5TGM1 cells. (**A**) Identification of upregulated (top) and downregulated (bottom) genes (left), and gene ontology analysis (right). Gene ontology results from GSEA were plotted as negative Log of P value for gene set enrichment and color coded for involvement in intracellular membrane or protein trafficking (pink), synthesis and secretion of secretory proteins from co-translational entry in the Endoplasmic Reticulum (ER) to the Golgi apparatus (orange), signaling (light blue), cell adhesion and motility (blue), metabolism (light green), cell death (red), cytoskeleton (green), and mitochondria (purple). (**B**–**D**) univariate analysis of expression of adhesion molecules (**A**) integrins, (**B**) cadherins, (**C**) intercellular adhesion molecules *p < 0.05, **p < 0.01, ***p < 0.001, ****p < 0.0001, ns non significant.

To assess the intrinsic role of VLA4 to tumor signaling, we deleted the alpha 4 (*α*_4_, *Itga4*) subunit in the WT 5TGM1-GFP murine myeloma cell line using CRISPR/Cas9 technology (**methods**). Briefly, guide RNA (gRNA) was used to make the KO clones that had at least 3 base pair (bp) of mismatch between the target site and any other site in the genome. The gRNAs were designed to mitigate the risk of off-target editing^30^. We confirmed deletion and assessed signaling pathways altered by deletion of *Itga4*, using RNAseq on the parental WT 5TGM1-GFP line and two independent CRISPR KO clones (KO1 and KO2) (Fig. 1). Globally, RNAseq showed 6553 and 6418 genes differentially regulated with *p* < 0.02 in clones KO1 and KO2, respectively, relative to WT 5TGM1. Overall, 6834 and 6678 genes were differentially expressed (DE) with *p* < 0.05 and q < 0.05 in the two sets. As expected, in both clones *Itga4* was significantly depleted (*P* < 1E-4) relative to WT matched samples; instead, integrin *β*_1_ (*Itgb1*) was slightly but significantly upregulated (Log2 FC 0.41–0.58, P = 5E-5) in both KO cell lines. Among DE genes with Log2 FC > 0.3 and *P* < 0.02, the 819 and 1447 genes that were upregulated vs downregulated in both KO clones relative to WT were selected for gene set enrichment analysis (GSEA, Molecular Signatures Database (MSigDB)). Upregulated genes overlapped sets involved in the function and structure of the endoplasmic reticulum (99 genes, − LogP > 20) and Golgi apparatus (− LogP > 16), protein and carbohydrate metabolism (− LogP > 14), regulation of cell death (93, − LogP > 16), cell adhesion (− LogP > 15), and motility (-LogP > 17). Downregulated gene sets included mitochondria structure and function (142, − LogP > 24), and the cytoskeleton (126, − LogP > 23). Signaling and intracellular trafficking gene sets showed upregulation or downregulation, reflective of changes in activation. Among others, both KO1 and KO2 clones upregulated 95 genes targeted by the transcription factor Nab2 (NGFI-A-binding protein 2, − LogP > 23), 101 genes (− LogP > 18) by Nrf2 (Nuclear factor erythroid 2-related factor 2)^31^, 75 targets of ETS2 (Protein C-ets-2, − LogP > 19), and 75 of NFY (Nuclear transcription factor Y, − LogP > 17). Among genes downregulated in KO cells, 149 were targets of NFAT (Nuclear factor of activated T-cells, -LogP > 19); 144 of these were also targeted by AP4 (adaptor protein complex 4, − LogP > 27), 99 by ERR1 (Estrogen-related receptor alpha, − LogP > 18), and 122 by PAX4 (Paired box protein-4)^32,33^. Targets of Foxn3 (Forkhead box protein N3, 118 genes, − LogP > 16)^34^ and Lef1^35^ (Lymphoid enhancer-binding factor 1, 97 genes − LogP > 13), including *Wnt4*, were downregulated. The other top transcription factor genes downregulated in KO cells were HMG20B (SWI/SNF-related matrix-associated actin-dependent regulator of chromatin subfamily E member 1-related, 131 genes, − LogP > 14,) and ZNF92 (Zinc finger protein 92, 112 genes, − LogP > 15) (FDR q < 1E-10)^36,37^ (Supplementary Fig. 1A, B). Univariate analysis of genes involved in cell adhesion was performed, showing high reproducibility across RNAseq WT samples and similarities across the two KO clones. Other integrin subunits were expressed, and KO MM cells (MMC) increased *Itgal*, *Itgb1*, *Itgb2*, *and Itgb5*. Despite their role in myeloma and bone cancer, neither *α*v*β*3 nor *β*7 were altered (Fig. 1B). *Cadh11* (osteoblast-cadherin) and *Cdh19* did not show significant differences, but *Cdh2* (N-cadherin), involved in homophilic interactions with bone marrow stromal cells (BMSC), by contrast, was significantly increased in KO clones (Fig. 1C). Also in the intercellular adhesion molecule (ICAM) family, myeloma-associated ICAM1 was upregulated in the KO MM clones (Fig. 1D). Overall, these data show that loss of *Itga4* produced significant changes in the transcriptome of myeloma cells, affecting functions classically associated with integrins, such as adhesion to the extracellular matrix, cell motility and signaling, but also the structure, metabolism, and homeostatic balance of the cell.

### In vitro phenotypic characterization of ***Itga4*** (α_4_) KO (VLA4-null) 5TGM1-GFP

**Figure 2:**
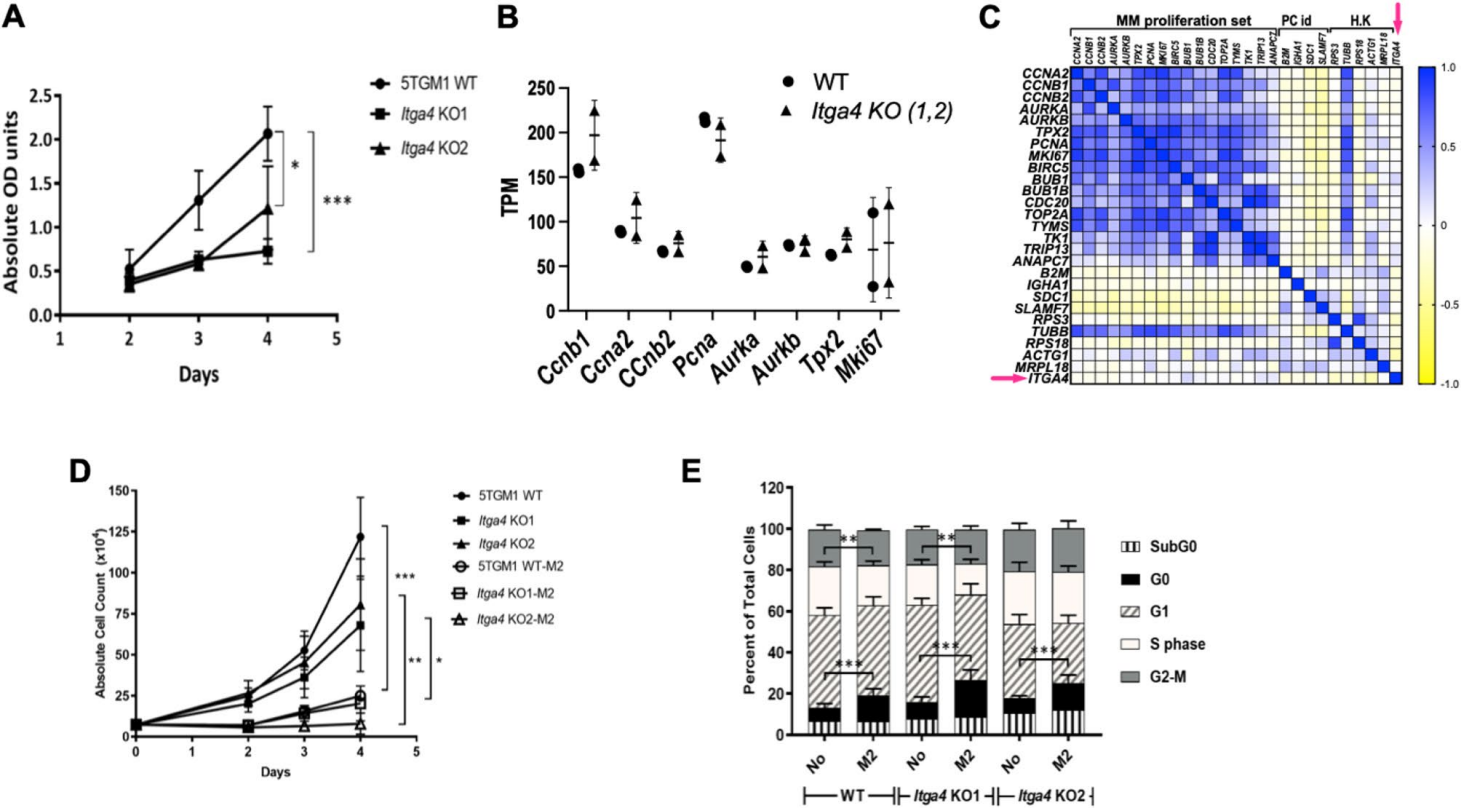
In vitro characterization and functional assessment of *Itga4* KO 5TGM1-GFP cells compared to WT 5TGM1-GFP cells. (**A**) The WST-1 proliferation assay comparing the WT with the KO cells. (**B**) gene expression by RNAseq of proliferation-associated genes in 5TGM1 WT and Itga4 KO clones KO1 and KO2. (**C**) Correlation matrix from RNAseq data from patient myeloma cells comparing ITGA4 *(*arrow) to the proliferative MM signature genes, plasma cell identity markers, and common housekeeping genes. (**D**) Fluorescence bead count assay to quantify proliferation of *Itga4* KO 5TGM1-GFP cells vs WT 5TGM1-GFP cells in the presence and absence of M2-10B4 (M2) stromal cells. The open and dense labels refer to cells cultured with and without M2-10B4 (M2). **(E)** Cell cycle assay comparing the *Itga4* KO 5TGM1-GFP cells vs. WT 5TGM1-GFP cells in the presence and absence of M2-10B4 (M2). Data are shown as mean and SD. All cell biology panels represent the combination of three independent experiments. (*p < 0.05, **p < 0.01, ***p < 0.001; unpaired t test).

To determine if deletion of *Itga4* affected proliferation of 5TGM1-GFP cells, we first compared cell growth of *Itga4* (α_4_) KO 5TGM1-GFP clones (KO1 and KO2) to WT 5TGM1-GFP cells by the Cell Proliferation Kit II (XTT) assay. Both KO clones had decreased formazan fixation, suggestive of reduced proliferation, relative to the VLA4 WT cells on day 4 (*p* < 0.001 (KO1) and *p* = 0.024 (KO2) (Fig. 2A). Dedicated analysis of the expression of genes involved in proliferation, however, showed no differences between *Itga4* WT and KO 5TGM1. In order to test the association between *Itga*4 and proliferation in human myeloma, we analyzed publically available datasets from the gene expression omnibus (GEO). The proliferation-associated gene signature for MM described by Hose et al.was used as reference for transcripts upregulated in quickly replicating MM^38^. In the GSE159426 dataset (RNAseq data from 53 MM patients), multivariate analysis compared the expression of *Itga4* relative to all of the 17 proliferation-signature transcripts^39^. Controls were chosen among MM markers, such as b2 microglobulin and CS1, and established housekeeping genes, such as tubulin or RPS18. Multivariate analysis showed strong positive correlation amongst the proliferation-associated genes, but no correlation with *Itga4* (Fig. 2C). Lack of correlation of ITGA4 with cyclin 2A and 1B was further validated in the GSE68871 dataset (Supplementary Fig. 1).

Upon cell–cell interaction with healthy stromal cells, MM cells assume characteristics of quiescence, reducing their proliferation rate. In order to experimentally address whether loss of *Itga4* prevented KO MM cells from interacting with the stroma, as well as more carefully characterize growth rate, we tested the interaction between BM stromal and the *Itga4* KO 5TGM1-GFP cells using the M2-10B4 murine BM stromal cell line, characterized by high fibronectin secretion in vitro^40^. Interestingly, in the presence of M2-10B4 stromal cells, both 5TGM1 WT and the KO clones had decreased cell counts, independent of α_4_ expression. Mean viable WT, KO1, and KO2 cell counts were reduced 4.87-fold (*p* < 0.001), 3.33-fold (*p* = 0.019), and 10.11-fold (*p* = 0.002), respectively (Fig. 2D). The absolute cell counts between the WT and KO cells in the absence of stroma were not significantly different (Fig. 2B). Accordingly, cell cycle analysis showed that the presence of stromal cells did not induce any difference in the fraction of cells in G1 and G2-M (Fig. 2E). Significant differences were observed primarily between “stroma” versus “no stroma”. Furthermore, the non-significant difference in cell counts between WT versus KO in Fig. 2B is corroborated by the lack of significant differences in the cell cycle analysis in Fig. 2E. There was no detected difference in cell apoptosis as implied by the sub G0 cell population. These results show that VLA4 expression is not indispensable for myeloma cells to be induced into quiescence. Gene expression, cell cycle analysis, and cell number evaluation over time in 5TGM1 clones further suggest that there may be no direct link between *Itga4* and proliferation, which is also reflected in human data.

### Deletion of VLA4 in 5TGM1 cells altered tumor localization to extramedullary sites in vivo contributing to enhanced survival

**Figure 3:**
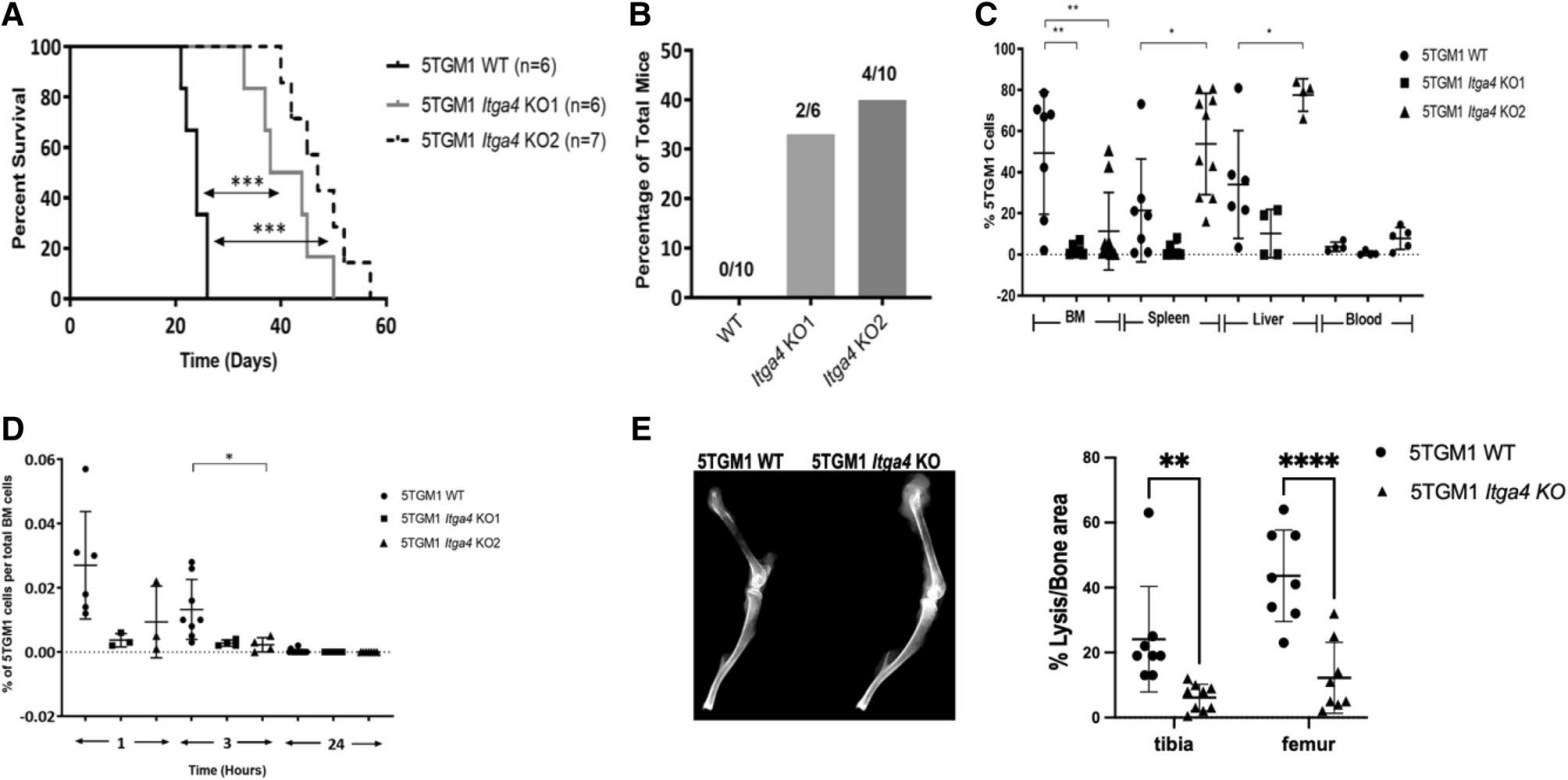
The in vivo characteristics of the *Itga4* KO 5TGM1-GFP cells were compared with the WT 5TGM1-GFP cells in the intravenous (i.v.) KaLwRij mouse model. (**A**) The *Itga4* KO 5TGM1-GFP cells engrafted in immunocompetent C57BL/KaLwRij mouse model demonstrated significant survival advantage. (**B**) Quantitation of number of mice that developed extramedullary tumors detected in mice engrafted with WT or *Itga4* KO 5TGM1-GFP cells. (**C**) Flow cytometry was used to measure percent 5TGM1 cell levels in different tissues. Each symbol represents a unique mouse. (**D**) Lack of *Itga4* in 5TGM1 cells led to reduced BM homing in in vivo competitive homing experiment (n = 18). Shown is the percentage of 5TGM1 cells in the BM. All graphs represent data at time of death. (**E**) Assessment of myeloma bone disease three weeks from inoculation of 5TGM1 WT vs *Itga4* KO, representative X-ray (left) and quantification of hypointense areas/total bone area in tibia and femur (N = 5 mice/group).

KaLwRij mice are a spontaneously generated, extensively characterized model of age-dependent monoclonal gammopathy. 5TGM1 cells, originating from a KaLwRij myeloma mouse and stably expressing GFP, are well characterized for their ability to engraft to the BM and spleen of KaLwRij mice establishing an aggressive form of MM, including paraproteinemia and bone disease, which is lethal within a month from inoculation^41^. To determine the role of VLA4 in tumor engraftment and dissemination, we injected 5TGM1 WT and KO clones intravenoustly (i.v.) and followed mice for survival and surrogate end-points (bilateral hind limb paralysis and severe cachexia). Mice engrafted with either *Itga4* (α_4_) KO 5TGM1-GFP clones demonstrated statistically significant prolonged survival compared to mice engrafted with WT 5TGM1-GFP. The median survival of mice with WT, KO1, and KO2 was 24, 41 (*p* < 0.001), and 47 days (*p* < 0.001), respectively (log-rank test; Fig. 3A). Both *Itga4* (α_4_) KO 5TGM1-GFP clones KO1 and KO2 demonstrated increased propensity (37%, 6/16) to form extramedullary plasmacytomas in the skin, subcutaneous, and intraperitoneal areas (Fig. 3B). KO2 also showed increased tumor burden in the spleen and liver relative to the WT 5TGM1-GFP group (Fig. 3B). Average liver weight of KO2 and WT tumor bearing mice at week three was 1.98 g (± 1.39) and 1.24 g (± 0.11), respectively. No extramedullary plasmacytomas were observed in mice injected with WT 5TGM1-GFP cells (Fig. 3B). Further, analysis of BM harvested at the time of euthanasia or death showed that both KO1 and KO2 tumor mice had significantly lower MM cells in the BM compared to the WT tumor cells. The mean percentage of WT, KO1, and KO2 cells in the BM measured using flow cytometry was 54.78%, 2.42% (*p* = 0.001), and 15.37%, (*p* = 0.016) respectively (Fig. 3C).

VLA4 has physiological functions in lymphoid cell homing and participates in the vicious cycle of osteolysis in myeloma bone disease. Having found lower myeloma cell prevalence in the BM of mice injected with *Itga4* KO 5TGM1 cells, we set out to evaluate homing and osteolysis in 5TGM1 KO MM.

Less number of *Itga4* (α_4_) 5TGM1-GFP KO2 cells were detected in the BM than 5TGM1-GFP WT cells after 15 and 30 min of i.v. tumor injection (*p* = 0.016 and *p* = 0.043, respectively) (Fig. 3D). Bone disease was evalueted three weeks after tumor inoculation, when young KaLwRij mice injected with 5TGM1 cells are known to show multifocal myeloma bone disease. Indeed, mice with WT 5TGM1 tumors presented severe osteolysis in both tibiae and femurs, which could be observed by *ex-vivo* radiography (Fig. 3E). By contrast, in *Itga4* KO MM mice, hypodense foci occupied a significantly smaller area of both tibia and femur (Fig. 3E).

In order to evaluate whether loss of VLA4 would have similar effects on human myeloma cells, *Itga4* (α_4_) CRISPR KO was performed on the human MM.1S cell line, an IgA lambda MM that establishes orthotopic myeloma xenografts when injected i.v. in nod scid gamma (NSG) mice. *Itga4* KO MM.1S cells in NSG mice showed decreased intramedullary tumor burden in the BM, and increased formation of extramedullary plasmacytomas relative to WT. Further, survival was significantly longer in mice injected with *Itga4* KO MM.1S cells (Supplementary Fig. 3). These results confirm the in vivo phenotype of *Itga*4 KO in a human cell line.

In all, these results show that ablation of VLA4 through deletion of the *ITGA4* (α_4_) subunit impairs myeloma cell homing and dissemination in the bone, prolonging survival and reducing osteolysis.

### NIR imaging with LLP2A-Cy5

**Figure 4:**
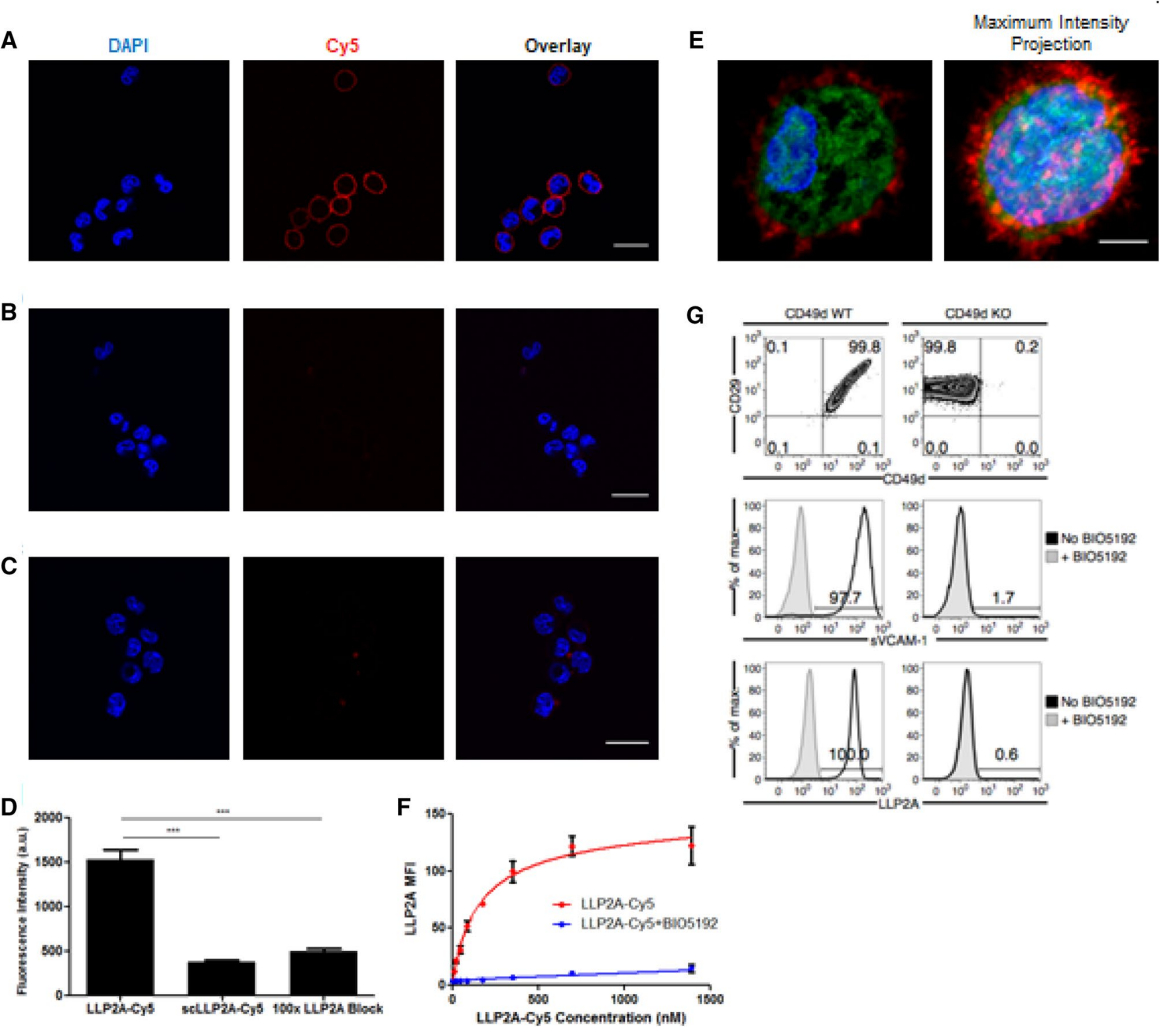
LLP2A-Cy5 specificity and affinity to VLA4 in vitro. (**A**) Strong cell surface fluorescence was observed in the WT 5TGM1-GFP cells incubated with LLP2A-Cy5 (1 µM, 15 min, at 4 °C). Blue, green and red colors represent nuclear stain, GFP, and Cy5 signal, respectively in all images (60x; scale bar: 20 µm). (**B**) Cell surface signal was decreased when the same was co-incubated with 100-fold excess of LLP2A-PEG4 blocking peptide (100 µM). (**C**) Cell surface binding was also reduced in the WT 5TGM1-GFP cells treated with scLLPA-Cy5 (1 µM, 15 min, at 4 °C). (**D**) Quantification of background corrected cell fluorescence showed significantly increased signal in LLP2A-Cy5 incubated cells, relative to scLLP2A-Cy5 and blocking (***p < 0.001 1-way analysis of variance (ANOVA) with Bonferroni multiple comparisons test). (**E**) Representative high-resolution imaging of a single cell incubated with LLP2A-Cy5 for 2.5 h at 37 °C. Cells exhibiting minimal motion at 60 × magnification were imaged in a confocal 3D z-stack. Post-deconvolution visualization of a single confocal plane (left) and maximal intensity projection (right) of the confocal 3D z-stack (100x; scale bar: 3 µm) show strong cell surface binding with minimal internalization of LLP2A-Cy5. (**F**) FACS of LLP2A-Cy5 incubated with WT 5TGM1-GFP cells demonstrated selective binding of LLP2A-Cy5 to VLA4. WT 5TGM1-GFP cells and varying LLP2A-Cy5 concentrations (0-1400 nM) were co-incubated for 30 min and washed with Tyrode’s buffer containing 1% BSA. BIO5192 (100 nM) was used to evaluate non-specific binding. (**G**) LLP2A-Cy5 showed minimal binding to VLA4 KO cells relative to VLA4^+^ WT tumor cells. (Top) 1.5 × 10^5^ WT and KO 5TGM1-GFP cells were assessed for *α*_4_ and *β*_1_ levels, and for binding with sVCAM-1 (Middle), and (Bottom) LLP2A-Cy5 respectively.

We have successfully shown nuclear imaging using the VLA4 peptidomimemic, LLP2A, and now sought to test the analogous NIR molecular imaging probe, LLP2A-Cy5, since it will allow for imaging on a cellular level as well as preclinical whole-body level. To this end, we evaluated LLP2A-Cy5 in vitro and in vivo. We found that the LLP2A-Cy5 construct demonstrated high binding affinity and specificity for activated VLA4 on the surface of the 5TGM1-GFP murine myeloma cells in vitro (Fig. 4A). LLP2A-Cy5 showed significantly reduced cell surface binding in the presence of the scrambled control (3.96 ± 0.32-fold reduction) and blocking with excess unlabeled peptide (3.07 ± 0.3-fold reduction) (Fig. 4B–D). Incubation at 37 °C for an extended period (2.5 h) showed dominant cell membrane binding (Fig. 4E). Binding to activated VLA4 was specific (K_d_: 148.4 ± 14.2 nM), with minimal nonspecific activity demonstrated in the presence of BIO5192, a synthetic inhibitor specific for activated VLA4^42^ (Fig. 4F). Finally, reduced uptake of LLP2A-Cy5 by the KO cell lines relative to WT cells validated the specificity of the LLP2A-Cy5 to VLA4 (Fig. 4G top). Soluble VCAM-1 (sVCAM-1) binding was reduced in the KO cells as well, indicating a loss of VLA4 activity (1.65 ± 0.07% vs. 97.7% in the WT; Fig. 4G middle), while LLP2A-Cy5 binding (0.6% vs. 100% in the WT; Fig. 4G bottom) was nearly eliminated. Pre-incubation with an established therapeutic dose of BIO5192 yielded similar activities for LLP2A-Cy5 (0.95% WT vs. 0.52% KO) and sVCAM-1 (3.33% WT vs. 2.51% KO). These results indicate the selective affinity of LLP2A-Cy5 for activated VLA4 in vitro. Next, six MM cell lines were evaluated using LLP2A-Cy5 by flow cytometry.

**Figure 5:**
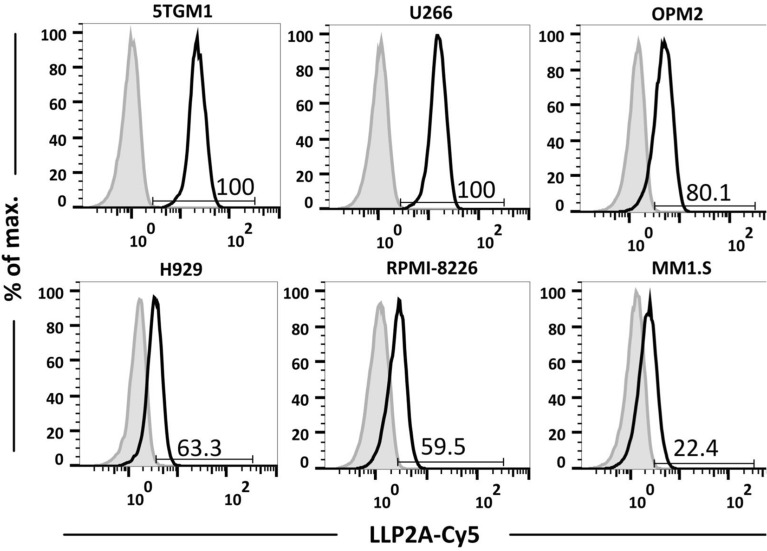
LLP2A-Cy5 binding to diverse MM cell lines. Flow cytometry was performed to evaluate binding of LLP2A-Cy5 to different myeloma cell lines.

The expression of activated VLA4 on MM cell lines varied as follows (% LLP2A-Cy5^+^ and LLP2A-Cy5^+^ median fluorescence intensity (MFI) in parentheses, respectively): 5TGM1 (100%, 23.4) > U266 (100%, 16.1) > OPM2 (80.1%, 4.6) > H929 (63.3%, 3.4) > RPMI-8226 (59.5%, 3.2) > MM1.S (22.4%, 2.1) (Fig. 5).

### LLP2A-Cy5 binds dimeric activated VLA4 in primary malignant plasma cells

Myeloma cells express multiple integrins, capable of forming different heterodimers. CD29, integrin beta 1, was previously shown to be associated with poorer prognosis in myeloma patients. The CoMMpass dataset was interrogated for variability in the expression of *Itga4* and its association with common cytogenetic abnormalities. Gene expression of *Itga4* was usually present, though its levels were variable. Overexpression of *Itga4* was more prevalent in t(11;14) primary myelomas (Supplementary Fig. 2A). In cell lines, *Itga4* was expressed at high levels in t(11:14) positive KMS12 line (Supplementary Fig. 2B)^43^, but also negative in MM.1S (Supplementary Fig. 3).

**Figure 6:**
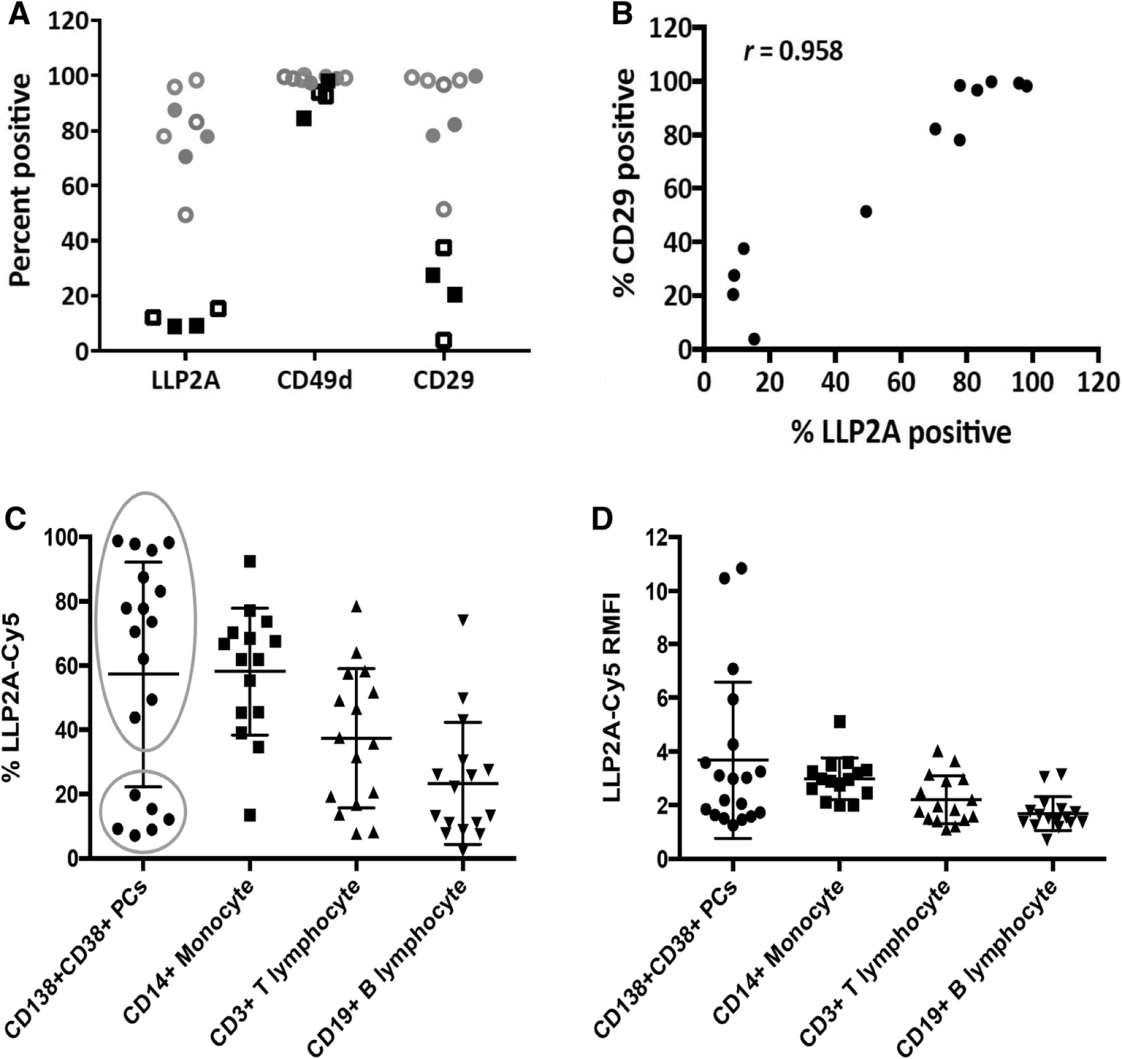
LLP2A-Cy5 reports on expression of dimeric VLA4 in primary human multiple myeloma. (**A**) Percentage of LLP2A + , CD49d + (*α*_4_) and CD29 + (*β*_1_) on CD138 + CD38 + cells from 12 MM BM patient samples. Dense and open symbols referred to newly diagnosed and relapsed patient samples, respectively. (**B**) The correlation between percentage of LLP2A + cells and CD29 + cells [Pearson correlation coefficient with r = 0.958 (*p* < 0.0001)]. (**C**) The percentage of LLP2A + cells and (**D**) relative mean fluorescent intensity (RMFI) on different cell types (19 MM PC samples).

Integrin transcription is tightly regulated, and responsive to differentiation and environmental signals. Integrin function, however, depends largely on post-translational events: heterodimerization, exposure on the cell membrane, and inside-out or outside-in conformational changes regulating its active or inactive state. In the lymphoid lineage, besides VLA4, integrin beta1 (CD29) can form VLA5 in complex with α_5_, and α_4_ (CD49d) can bind β_7_ forming LPAM—both described in myeloma. LLP2A was shown to specifically bind to the activated form of VLA4, disambiguating the significance of CD29 and CD49d positivity. Therefore, LLP2A-Cy5 was tested on nineteen de-identified BM samples from myeloma patients and compared to the VLA4 subunits, CD29 and CD49d. MM plasma cells (PCs) were identified by positive staining to CD138 and CD38. In this population, CD49d was expressed by over 80% of cells in all samples, CD29 was over 20% in 94% of the patients, and LLP2A-Cy5 stained over 20% of cells in 68% (13/19) of CD138 + CD38 + MM PCs (Fig. 6A). Interestingly, binding of LLP2A-Cy5 correlated linearly with CD29 expression (r = 0.958, *p* < 0.001, Fig. 6B), suggesting that the β1 subunit might be the limiting monomer for VLA4 formation. Looking into specificity of binding to MM PC, staining panels were used to identify monocytes, B and T lymphocytes. LLP2A-Cy5 was found to bind these populations as follows: monocytes (58.2%), T-lymphocytes (36.3%), and B-lymphocytes (23.3%) (Fig. 6C). Analysis of fluorescence intensity showed that relative median fluorescence (RMFI) was 3.0 in monocytes, 2.2 in T-lymphocytes, and 1.7 in T cells (Fig. 6D). Overall, these results show that in tissues from myeloma patients, LLP2A-Cy5 staining provided non-redundant information on the expression and activation of VLA4.

### In vivo and ex vivo NIR imaging using LLP2A-Cy5

Noninvasive fluorescence imaging showed strong co-localization of LLP2A-Cy5 with medullary tumor GFP signal 18 h post-injection, indicating high tumor uptake in the immunocompetent 5TGM1/KaLwRij intravenous (i.v.) MM model (Supplementary Fig. 4A). Overall, LLP2A-Cy5 signal demonstrated rapid distribution and selective retention in the tumor at 4-h post-injection, while contrast peaked by ~ 18 h post-injection (Supplementary Fig. 4A). Fluorescence was highest in the BM (femur-muscle ratio, 7.0 ± 2.5; *p* < 0.01) and spleen (spleen-muscle ratio, 2.6 ± 0.7; *p* < 0.001), corresponding to tumor burden (Supplementary Fig. 4B). Fluorescence-activated cell sorting (FACS) of ex vivo BM samples confirmed the high specificity of LLP2A-Cy5 for VLA4 expressing cells in the BM (84.7% LLP2A-Cy5^+^; 97.5% VLA4^+^), spleen (83.7% LLP2-Cy5^+^; 91.8% VLA4^+^) (Supplementary Fig. 3C–D), and the hematopoietic BM niche (63.6% VLA4^+^, 53.2% LLP2A-Cy5^+^). Together, in vivo and ex vivo results indicated high specificity of LLP2A-Cy5 for VLA-4 overexpression on MM cells within the BM microenvironment that is maintained throughout FACS processing and analysis, providing evidence of potential utility for human diagnostic applications^44^.

**Figure 7:**
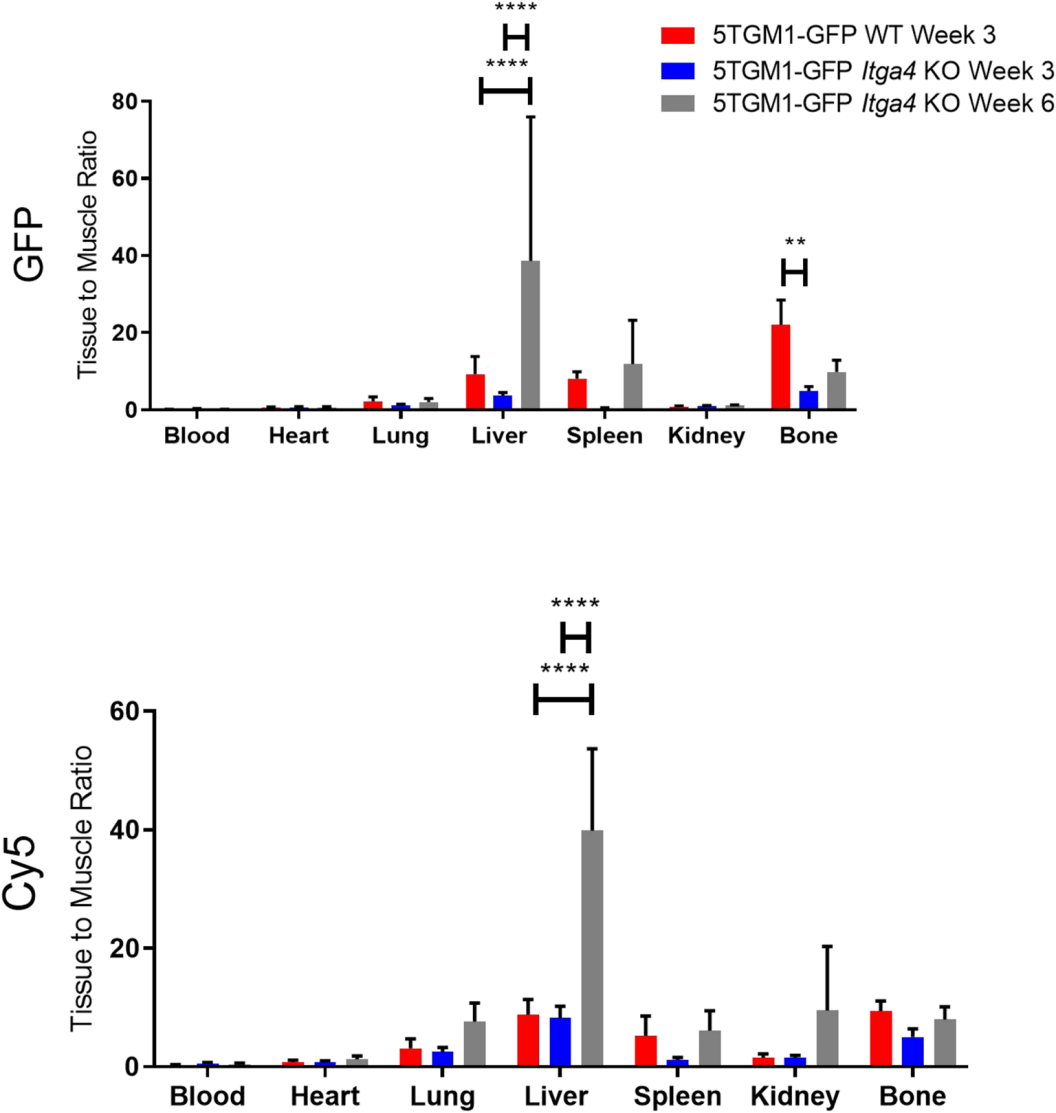
LLP2A-Cy5 NIR imaging of C57Bl/6 KaLwRij mice injected with 5TGM1-GFP WT and KO cells. Normalized biodistribution [defined as tissue to muscle ratio (TMR)] of GFP and Cy5 three and six weeks post-administration of cells. Two-way ANOVA followed by Sidak’s multiple comparisons test was performed on biodistribution data (**p < 0.01; ****p < 0.0001).

We further evaluated the VLA4-selective uptake of LLP2A-Cy5 in an in vivo study where we injected 1 × 10^6^ WT 5TGM1-GFP and *Itga4* (α_4_) KO 5TGM1-GFP (KO2) cells intravenously in 10 week-old female KaLwRij mice (N = 10/cohort). At three weeks post inoculation of the tumor cells, mice were injected with 25 μM (0.18 mg/kg) LLP2A-Cy5 for ex vivo fluorescence imaging and histology. Ex vivo imaging results confirm low tumor burden in the BM of KO tumor mice as compared to the WT mice at week three. While the WT 5TGM1-GFP tumor bearing mice were euthanized at week three when there was terminal disease burden, the *Itga4* (α_4_) KO 5TGM1-GFP mice were euthanized at week three and week six respectively for ex vivo tissue bio distribution (Fig. 7). The ex vivo images support the tissue bio distribution data (Supplementary Fig. 5) and the histological data confirm tumor in the liver of KO mice (Supplementary Fig. 6). We also confirmed the presence of viable tumor cells in both the mouse models by serum protein electrophoresis (SPEP) assay. Quantification of SPEP data revealed that at week three, high percent of M-protein (γ-globulin) was present in serum samples of 5TGM1-GFP WT mice (5.52 ± 1.65%) as compared to *Itga4* (α_4_) KO 5TGM1-GFP mice (2.80 ± 1.10%). Although, the percentage γ-globulin in serum samples of *Itga4* (α_4_) KO 5TGM1-GFP mice increased to 5.71 ± 1.03% at week six (Supplementary Fig. 7. This SPEP data correlates with the median survival as shown in Fig. 3A (24 days for 5TGM1-GFP WT mice, 42 and 47 days for *Itga4* (α_4_) KO1 and KO2 5TGM1-GFP mice, respectively).

## Discussion

VLA4 (CD49d/CD29, α_4_β_1_) is a noncovalent, heterodimeric transmembrane receptor that plays an instrumental role in myeloma pathogenesis. The significance of VLA4 in homing, proliferation and drug resistance in MM pathogenesis is extensively supported by literature. The cross talk between the conformationally active VLA4 on the MM cells and VCAM-1 expressed on BM stromal cells (BMSCs) aggravates bone destruction^3,45^. Studies have confirmed the presence and correlation of VCAM-1 with advanced disease features in MM patients^46,47^, and activated CD29 (β_1_) has been verified on myelomatous BM plasma cells^48–50^. Furthermore, the amplification of VLA4 on drug resistant MM cells makes it a logical target for eradicating resistant clones, as recently shown in preclinical nanotherapeutic targeting experiments^24^.

Here we investigated the effect of VLA4 on in vivo trafficking and survival in a controlled preclinical setting. We report for the first time, two VLA4-null MM 5TGM1 KO clones generated by CRISPR/Cas9 knockout of the *Itga4* (α_4_) subunit. RNAseq performed on the CRISPR KO clones showed differentially regulated genes relative to the WT 5TGM1 parental cells. The loss of *Itga4* produced significant changes in the transcriptome of myeloma cells, affecting functions classically associated with integrins, such as adhesion to the extracellular matrix, cell motility and signaling, but also the structure, metabolism, and homeostatic balance of the cell. Flow cytometry and fluorescence microscopy validated the absence of VLA4 in the KO cell lines. Next, we utilized the 5TGM1 WT and KO cells in the immunocompetent syngeneic KaLwRij mouse model to investigate the impact of VLA4 modulation on myeloma pathogenesis. Notably, the lack of α_4_ diminished BM tumor burden, while there was increased propensity to form extramedullary plasmacytomas. The overall survival was prolonged in the *Itga4* (α_4_) KO 5TGM1-GFP/KaLwRij mouse model. Accordingly, univariate analysis of selected genes (Fig. 1B–D) shows overexpression, perhaps compensatory, of other adhesion molecules. Relative to the VLA4 WT cells, the *Itga4* (α_4_) KO cells overexpressed integrins alpha(5) and beta (1), and alpha(L) beta (2). Similarly to VLA4, VLA5 (α_5_β_1_) can bind fibronectin, and LFA-1 (α_L_β_2_) is a known mediator of CAM-DR. KO cells also overexpressed N-cadherin, expressed by osteogenic precursors, and ICAM1, also implicated in adhesion and CAM-DR. Furthermore, reduced osteolysis in the KO tumor bearing mice as opposed to the significantly higher BM engraftment of the WT tumors likely contributes to the prolonged overall survival^14,51,52^. The in vivo phenotypic studies of *Itga4* (α_4_) KO 5TGM1-GFP and WT 5TGM1-GFP, indicates that VLA4 on MM cells participates in BM homing, extramedullary manifestation, BM tumor burden, and survival, emphasizing the importance of MM-BM microenvironment interaction contributed by VLA4.

VLA4 as a therapeutic has been evaluated preclinically and clinically. Natalizumab is a humanized monoclonal antibody^53^, designed to target VLA4 on the surface of leukocytes and has been approved as a disease-modifying therapy for patients with relapsing remitting multiple sclerosis^54,55^. Direct targeting of VLA4 has yielded promising results in a mouse model melanoma^56,57^. Nair-Gupta et al. demonstrated that blockade of VLA4 sensitized leukemic and myeloma cells to CD3 redirection in BM, highlighting VLA4 targeting as a viable combination therapy target^58^. There is a strong premise for the evaluation of VLA-4 as an imaging and therapeutic target in MM.

We validated the VLA4 targeted NIR probe, LLP2A-Cy5, for imaging surface-level, activated VLA4 expression in vitro and in vivo. The LLP2A-Cy5 construct demonstrated high binding affinity and specificity for activated VLA4 on the surface of the 5TGM1-GFP murine myeloma cells in vitro. The cell studies indicated a lack of internalization at steady state, with NIR fluorescence primarily localized at the tumor cell surface. VLA4 has a longer cell surface exposure relative to other integrins^59^, which suggests that the fluorescence remains trapped at the cell surface due to constant integrin recycling with minimal uptake of the LLP2A-Cy5 in the endosomes. The lack of NIR fluorescence in endosomes or the cytoplasm suggests an antagonist mechanism for LLP2A-Cy5, since ligand-based activation of integrins results in endocytosis and release of ligands into the endosome prior to integrin recycling^60^.

In vivo optical imaging of myeloma using bioluminescent reporters and xenograft models has provided insights into VLA4 expression, survival and tumor cell homing^26,61,62^. In our studies, LLP2A-Cy5 showed selective VLA4 binding in tumor tissue compared to non-tumor VLA4 expressing tissue by FACS analysis; while overall spleen and BM uptake of LLP2A-Cy5 was significantly higher in the WT 5TGM1-GFP tumor bearing mice. The 2D GFP and Cy5 (LLP2A-Cy5) ex vivo images of excised bone and non-tumor tissue from 5TGM1-GFP WT and 5TGM1-GFP KO mice show the spatial distribution of the 5TGM1-GFP cells in various tissues. These images combined with normalized tissue biodistribution of GFP and LLP2A-Cy5 at three weeks post-administration of tumor cells confirm the biological preference of VLA4^+^ WT 5TGM1-GFP cells to engraft in the BM. The biodistribution of the KO cells in the KaLwRij mouse model was vastly different with preferential uptake in the extramedullary organs such as liver, spleen over BM. These results demonstrate the comparable viability in vivo and in vitro of LLP2A-Cy5 to previously studied nuclear LLP2A tracers. Furthermore, these results affirm the complementary use of non-ionizing NIR molecular imaging probes for imaging cells and tissues in a non-destructive and longitudinal fashion in preclinical MM. Of relevance to the clinic, we showed staining with LLP2A-Cy5 of nineteen de-identified myeloma samples from patients. Specifically, the assay demonstrated that high levels of LLP2A-Cy5 binding were associated with high expression of both β_1_ and α_4_, while the reverse was not equally true; pointing to non-redundancy of the information that could be obtained with LLP2A-Cy5 staining. As expected for an integrin involved in migration of a number of leukocyte subset, we noted staining with LLP2A-Cy5 of non-tumor hematopoietic cells. Among those, the highest staining was observed in bone monocytes, which in myeloma assume pro-osteoclastogenic and pro-tumor characteristics^63^, and we have previously shown increased LLP2A-Cy5 binding exclusively in the presence of MM^28^.

In summary, here we generated *Itga4* (α_4_) KO MM cells and evaluated their biological activity in vitro and in vivo*.* Significant differences in the transcriptome were noted between the WT and KO clones. Biologically, mice bearing *Itga4* (VLA4) KO tumors demonstrated an enhancement in survival likely due to differential tumor cell distribution in vivo*.* The in vitro and in vivo characterization was investigated using the exogenous NIR molecularly targeted VLA4 specific imaging agent, LLP2A-Cy5. LLP2A-Cy5 was instrumental in establishing the spatial localization of medullar myeloma lesions in the syngeneic immunocompetent 5TGM1/KaLwRij mouse model. Utilization of immunocompetent syngeneic mouse model is a strength and core focus of this work. In a proof-of-principle experiment, we additionally showed that *Itga4* (α_4_) KO in the human MM cell line, MM.1S, similarly led to decreased intramedullary tumor burden in the BM, and formation of extramedullary plasmacytomas relative to WT. Survival was also significantly longer in mice injected with *Itga4* KO MM.1S cells.

As new therapies for MM are being developed for generating deep remissions and long-term survival, we anticipate that the functional significance of integrins such as VLA4 will be key to understanding and modulating therapeutic pathways. The data reported here support the continued development of VLA4 targeted molecular imaging and therapeutic agents for MM with particular attention to potential off-tumor and off-target toxicities. Our future studies will test the hypothesis that the VLA4 KO cells can be sensitized to a wide range of myeloma therapies, as these cells demonstrate impaired binding to the protective BM environment in vivo.

## Materials and methods

### Animal studies

All methods are reported in accordance with ARRIVE guidelines. All animal studies were performed in accordance with the Guide for the Care and Use of Laboratory Animals under the auspices of the Animal Studies Committee of Washington University (Animal Welfare Assurance number—D16-00,245).

### Statistical analysis

All LLP2A-Cy5 imaging data is presented as mean + standard deviation and statistical analysis was performed using GraphPad Prism Version 9.1.0 software. Statistical significance on the biodistribution data was calculated using two-way analysis of variance (ANOVA) followed by Sidak’s multiple comparisons test, unless specified otherwise. P values of less than 0.05 were considered statistically significant.

### Cell culture and Reagents

WT 5TGM1-GFP cells (kind gift from Dr. G. Mundy, Vanderbilt University, Nashville, TN, USA) were maintained at 10^6^ cells/mL in Iscove’s Modified Dulbecco Medium supplemented with 10% v/v fetal bovine serum and 1% v/v penicillin/streptomycin (all from Thermo Fisher Scientific, MA). LLP2A-PEG4-Cy5 (LLP2A-Cy5) and scrambled LLP2A-PEG4-Cy5 (scLLP2A-Cy5) were synthesized as previously described^28^ and purchased from Auspep (Tullamarine Victoria, Australia).

### Confocal microscopy

WT 5TGM1-GFP cells were plated on 35 mm glass bottom MatTek dishes pre-coated with poly-D-lysine (glass no. 1.5; MatTek Corporation, MA) at 10^5^ cells/cm^2^ and allowed to attach for 1 h at 37 °C. Cells were incubated with the Cy5-labelled probes (1 µM) at 4 °C for 15 min, and at 37 °C for 2.5 h to assess cell surface binding specificity and cellular internalization, respectively. Hoechst 34,580 (10 µg/L, Invitrogen, CA) was added for the last 15 min of incubation to counterstain cell nuclei. Fluorescence confocal microscopy was performed on a Nikon A1Rsi confocal microscope (Nikon Corporation, Tokyo, Japan) with 60X and 100X oil immersion objectives using excitation-emission settings at 405 nm-450/40 nm (Hoechst 34,580), 488 nm-530/20 nm (GFP), and 640–660/20 nm (Cy5).

### Saturation binding assay

Selective affinity was determined by fluorescence activated cell sorting (FACS). 1.5 × 10^5^ WT 5TGM1-GFP cells were incubated with varying concentrations of LLP2A-Cy5, with or without 100 nM BIO5192 (Tocris Bioscience, Bristol, United Kingdom). Samples were analyzed on a Beckman Coulter Gallios flow cytometer and data were analyzed using FlowJo software (TreeStar, Ashland, OR). Total and nonspecific binding coefficients (dissociation constant (K_d_) and receptor density (B_max_)) were calculated by fitting mean fluorescence intensity (MFI) versus LLP2A-Cy5 concentration using Prism 5.0 (GraphPad Software, Inc., CA).

### Binding studies with MM patient samples

All patient samples and data were collected and analyzed under the Institutional Review Board Washington University and Declaration of Helsinki protocols. The CD138 positive and negative populations from MM (multiple myeloma) patient BM (bone marrow) were sorted by autoMACS pro separator using human CD138 microbeads and indirect human CD34 microbead kit (Miltenyi Biotec, Germany), respectively. Briefly, CD138 + cells from the bone marrow of MM patients were purified by immunomagnetic selection using an automacs device. Purified cells were > 97% for CD38 + CD138 + plasma cells. LLP2A-Cy5 staining for flow cytometry was done similar to standard antibody labeling procedures, except here we used Hanks Balanced Salt Solution (HBSS) containing Ca^2+^, Mg^2+^ and 0.1% BSA as the staining buffer. The negative control consisted of sample treated with 100 nM BIO5192 (VLA4 inhibitor; Tocris) at room temperature for 30 min and washed twice before LLP2A-Cy5 staining.

### Flow cytometry and reagents

The single cells were suspended in phosphate-buffered saline (PBS) with 0.1% bovine serum albumin as buffer. The human and mouse F_C_ receptors were blocked by human BD Fc block and mouse BD Fc block, respectively. The cell suspension was incubated with fluorochrome-conjugated monoclonal antibodies in dark, at room temperature, for 30 min. Cells were washed twice with buffer afterward, and then proceeded analysis on a Beckman Coulter Gallios flow cytometer. The dead cells were excluded by using 7-amino-actinonycin D (7-AAD). The data were analyzed on FlowJo software. The appropriate isotype antibodies were used as controls. The fluorochrome-conjugated monoclonal antibodies were used as follows; for human: CD138-BV421 (clone MI15, Biolegend), CD38-FITC (clone HB-7, BD), CD29-APC (clone TS2/16, Biolegend), CD49d-PE-CF594 (clone 9F10, BD), CD3-FITC (clone UCHT1, BD), CD19-BV421 (clone HIB19, BD), CD14-PE-CF594 (cloneMOP9, BD), CD45-BV510 (clone HI30, BD) and for mouse: CD138-BV421 (clone 281–2, BD), CD29-APC-eFluor780 (clone HMb1-1, eBioscience), CD49d-APC (clone R1-2, Biolegend), CD45-BV510 (clone 30-F11, Biolegend).

### In vivo binding affinity

All animal studies were conducted according to protocols approved by the Washington University Animal Studies Committee. Mice were anesthetized for all treatments and imaging with 2% v/v isoflurane/100% O_2_. Female 1–3 month-old C57Bl/6 KaLwRij mice were injected intravenously (i.v.) with 10^6^ 5TGM1-GFP cells in 100 µL 1 × PBS via the lateral tail vein. Imaging studies were performed 3–4 weeks after tumor implantation. Prior to imaging, hair was removed by gentle clipping and depilatory cream to improve light transmission. Supplemental Fig. S1. Mice were imaged before and 4 and 18 h after intravenous injection of 100 µL of 25 µM LLP2A-Cy5 in 5% v/v DMSO/H_2_O using the Optix MX3 time-domain diffuse optical imaging system (Advanced Research Technologies, Montreal, Canada) as previously described^64^. The images were background subtracted and gated by fluorescence lifetime to minimize autofluorescence and diet-related non-specific fluorescence^65,66^.

### Generating ***Itga4*** (α_4_) KO 5TGM1-GFP cell line by CRISPR-Cas9

5TGM1-GFP cells were transiently co-transfected with 1 µg of gRNA (5’ GGGGAGTCTATAGCGAATCT 3’) and 1 µg Cas9 expression plasmids via nucleofection (Lonza, 4D-NUCLEOFECTOR X-unit) using solution P3, program CA-137 in small cuvettes according to the manufacturers recommended protocol. The α_4_-KO cells were single cell sorted by flow cytometry, clonally selected, and verified for disruption of the endogenous locus via targeted deep sequencing to identify frameshift mutations.

### Library generation and alignment (GTAC) and RNAseq analysis

Two *Itga4* clones of KO 5TGM1 were compared for gene expression to two independent samples of 5TGM1 WT cells. For each pair, differentially regulated genes with a log2 fold change greater than 0.3 were ranked by P value to select the top 2500 genes upregulated in each clone. Gene lists were compared using Venny (https://bioinfogp.cnb.csic.es/tools/venny/index.html). The 819 genes up-regulated and 2500 genes downregulated in both KO cell lines were evaluated for ontology and function by GSEA with the molecular signature database^67,68^. Pathway representation was ranked by enrichment, graphed as negative logarithm of the P value, and color-coded for related functions (e.g. endoplasmic reticulum and Golgi apparatus relating to synthesis of secretory proteins). Transcription factor targeting analysis was performed on the GSEA MsigDB (NCI Informatics Technology for Cancer Research)^67^. Analysis of the overlap matrix for the most significantly enriched transcription factors was performed and the top 4 non- to little-overlapping transcription factor-based groups were represented as Venn diagrams using the Venny 2.1 tool (Oliveros, J.C. (2007–2015) Venny. An interactive tool for comparing lists with Venn's diagrams. https://bioinfogp.cnb.csic.es/tools/venny/index.html).

### Cell proliferation (The Cell Proliferation Kit II (XTT)) assay

The WT 5TGM1-GFP or *Itga4* KO 5TGM1-GFP cells were plated and cultured, in triplicate, in 96 well flat bottom plates with 1 × 10^4^ cells/100 μL/well in the RPMI media (supplement file). The proliferation assays were done as per manufacturer protocol using cell proliferation reagent WST-1 (Roche) with measurement of optical density (OD) unit at 450 nm. For evaluation of stromal cell effect, the 2.5 × 10^4^ cells of M2-10B4 stromal cells were plated overnight. At 24 h, the plates with and without stromal cells were incubated with 7.5 × 10^4^ of α_4_ WT or α_4_ KO 5TGM1-GFP cells in RPMI media. Prior to analysis, all cells were harvested from each well. The SPHERO AccuCount particles (IL, USA) were used for absolute cell count by flow cytometry.

### Cell cycle analysis

The *Itga4* WT or *Itga4* KO of 5TGM1-GFP cells were cultured with or without M2-10B4 stromal cells in 6 well plates for 3 to 4 days. 5TGM1 cells were then harvested via pipetting and incubated with a brilliant violet 421-labeled anti-CD138 antibody (clone 281–2, BD Biosciences) in the dark at room temperature for 30 min before washing twice with staining buffer (PBS supplemented with 0.5% bovine serum albumin and 2 mM EDTA). Intracellular staining with a fluorescein isothiocyanate (FITC)-labeled anti-KI67 antibody (clone SolA15, eBioscience) was performed with a Foxp3 transcription factor staining buffer set. Cells were treated with 100 μg/mL ribonuclease A (Sigma-Aldrich) for 15 min before incubation with propidium iodide (PI) in the dark for 10 min and analysis by flow cytometry. Samples were analyzed on a Gallios flow cytometer (Beckman Coulter, Brea, CA) and the proportions of cells in the G0 (KI-67^-^PI^lo^), G1 (KI-67^+^PI^lo^), S (KI-67^+^PI^int^), and G2/M (KI-67^+^PI^hi^) phases of the cell cycle were determined by evaluating KI-67 and PI expression in CD138 + 5TGM1 cells using FlowJo software (TreeStar, Ashland, OR).

### In vivo competitive homing experiment on 5TGM1/KaLwRij immunocompetent mouse model

Itga4 KO 5TGM1-GFP cells were labeled with violet proliferative dye 450 (VPD450) per manufacturers protocol. The 4 × 10^6^ of VPD450 labeled *Itga4* KO 5TGM1-GFP KO clone1 or clone2 were mixed with 4 × 10^6^ of non-labeled WT 5TGM1-GFP. The 8 × 10^6^ equally mixed WT 5TGM1-GFP with Itga4 KO 5TGM1-GFP cells were injected via tail-vein into C57BL/KaLwRij mice. Blood was collected at 15, 30, 60, and 180 min after injection. Mice were killed, and BM from both femurs and spine, and spleen were extracted at 1, 3, and 24 h after injection. Single cell suspension was analyzed for 1,000,000 events/sample on flow cytometer. The percent of viable 5TGM1-α_4_ WT and 5TGM1-α_4_ KO cells were determined by GFP without or with VPD450 expression. In addition, at 24 h after injection, 15 × 10^6^ BM cells were cultured in vitro. All BM cells were harvested and analyzed for viable 5TGM1-GFP cells/well on 7th day by flow cytometry analysis.

### In vivo 5TGM1/KaLwRij immunocompetent mouse model

All mouse experiments were performed in compliance with protocols approved by the Washington University Animal Welfare Committee. All preclinical methods are reported in accordance with ARRIVE guidelines. 10^6^ 5TGM1-α_4_ WT or KO cells were injected via tail vein into 6–11 week old KaLwRij mice. Mice were killed when MM related humane endpoint symptoms (paralysis, mass, cachexia, and sickness) occurred. At time of death, the BM from spine and both femurs, spleen, liver, blood and mass were harvested and processed into a single cell suspension. The 5TGM1 cells were analyzed by flow cytometry using GFP^+^CD138^+^ viable cell population.

### *Itga4* KO MM1.S-GFP cells and in vivo MM.1S/NSG immunocompromised mouse model

The *Itga4* KO MM1.S-GFP were generated by using CRISPR-Cas9. Briefly, the α_4_ knockout guide RNA sequence was cloned into the MLM3636 plasmid (Addgene). 250,000 MM.1S cells were resuspended in 20μL SF solution (Lonza), 1 μg gRNA plasmids and 250 ng Cas9-HF plasmid (Addgene # 43,945 pc3 CAS9hc) and electroporated using the Lonza 4-D Nucleofector DS-137 program (optimized for these cells, not shown). *α*_4_ negative cells were sorted using a MoFlow sorter.

Six to ten-week old NOD.Cg-Prkdcscid Il2rgtm1Wjl/SzJ (NSG) were used in all MM.1S mice experiments. Briefly, 500,000 MM.1S parent or alpha4 KO cells were injected i.v. into tail veins of mice. Tumors were allowed to grow for ~ 2–3 weeks and tumor progression was followed by bioluminescence imaging. Flow cytometry for CD138 (Biolegend) was used to assess percent MM.1S cells in multiple tissues. Flow was run on an Attune and analyzed using FlowJo (Treestar).

### Ex vivo and in vivo NIR imaging

Supplementary Fig. 4. Mice were anesthetized for all imaging with 2% v/v isoflurane/100% O_2_. Female (n = 2)1–3 month old C57Bl/6 KaLwRij mice were injected with 1 × 10^6^ 5TGM1-GFP WT in 100 µL PBS via the lateral tail-vein. In vivo 3D fluorescence imaging in Supplemental Fig. 3A was performed on IVIS Spectrum CT (PerkinElmer, Waltham, MA) with auto-exposure settings in the GFP wavelength (Ex./Em. 480 nm/520 nm) filter pair. 100 µL of 25 µM LLP2A-Cy5 was administered via lateral tail vein in 5TGM1-GFP WT (n = 2) and no tumor C57Bl/6 KaLwRij mice (female, n = 2) three weeks post tumor cell implantation.

Supplementary Fig. 5. Mice were anesthetized for all imaging with 2% v/v isoflurane/100% O_2_. Female 1–3 month old C57Bl/6 KaLwRij mice were injected with 1 × 10^6^ 5TGM1-GFP WT (n = 11) or KO (n = 12) cells in 100 µL PBS via the lateral tail-vein. 100 µL of 25 µM LLP2A-Cy5 was administered via lateral tail vein in 5TGM1-GFP WT (n = 11) and KO mice (n = 6) three weeks post tumor cell implantation and in a separate cohort of 5TGM1-GFP KO mice (n = 6) six weeks post-tumor cell implantation.

All mice were sacrificed 18 h post-administration of LLP2A-Cy5 for 2D and 3D ex vivo imaging in the GFP and Cy5 wavelength (Ex./Em. 640 nm/680 nm) filter pairs. 2D analysis and 3D in vivo and ex vivo reconstructions were performed using the Living Image 4.7.1 analysis software. Background fluorescence was subtracted automatically via the “Adaptive Background Subtraction” module within the Living Image 4.7.1 program. Analysis of 2D ex vivo fluorescent images was performed by measuring Total Radiant Efficiency (TRE) (photons/sec/cm^2^/sr)/(μW/cm^2^) from ROIs drawn around bone and muscle tissue in the GFP and Cy5 images. Tissue to muscle ratio was calculated by dividing TREs of tissue by TREs of muscle tissue of each respective mouse.

### X-ray imaging for measuring bone osteolysis

For assessment of osteolytic lesions, legs were dissected by removing all skin and muscle tissues, fixed in formalin 10% for 48 h and preserved in 70% ethanol until analysis. Digital contact radiographs (25 kV, 0.4 mA, and 7.5 s) were then taken using a Faxitron UltraFocus100 scanner (Faxitron Bioptics, Tucson, AZ, USA) and osteolytic areas were traced and measured using ImageJ software (National Institutes of Health, USA).

### Serum protein electrophoresis

Serum protein electrophoresis (SPEP) was performed to identify M-protein spikes in the WT 5TGM1-GFP and *Itga4* KO 5TGM1-GFP myeloma mouse models used in this study. Blood samples were collected from the mice by cheek bleed technique in serum separating tubes. The serum was separated from samples by centrifuging at 1000 rpm for 10 min. These serum samples were analyzed on a QuickGel Chamber apparatus using pre-casted QuickGels (Helena laboratories) according to the manufacturer’s instructions. The % gamma globulin values were quantified from the bands using ImageJ software (Maryland, USA). The data is presented as mean + /− standard deviation using GraphPad Prism 8.0 (La Jolla, CA, USA).

## Supplementary Information

Supplementary Information 1.

Supplementary Information 2.
