# Supplementary material for: Ablation of VLA4 in multiple myeloma cells redirects tumor spread and prolongs survival
Source: Sci Rep. 2022 Jan 7;12:30. doi: 10.1038/s41598-021-03748-0 (PMC8741970; doi:10.1038/s41598-021-03748-0)
Supplement: Supplementary file 1 — Supplementary Information 1. [file 41598_2021_3748_MOESM1_ESM.docx]

**Editorial office QC comment**: We note that the images of the original blots appear to be still closely cropped (Supplementary Figure 7). Are you able to provide images showing full-length membranes, with membrane edges visible, for this? Were the blots cut prior to hybridisation with antibodies? If original images of full-length blots cannot be provided, please include images of all blots as they are, with membrane edges visible, and for all replicates performed in the Supplementary Information file and include an explanation for the absence of images of adequate length where appropriate in the manuscript (i.e. methods and/or figure legends).

Response:


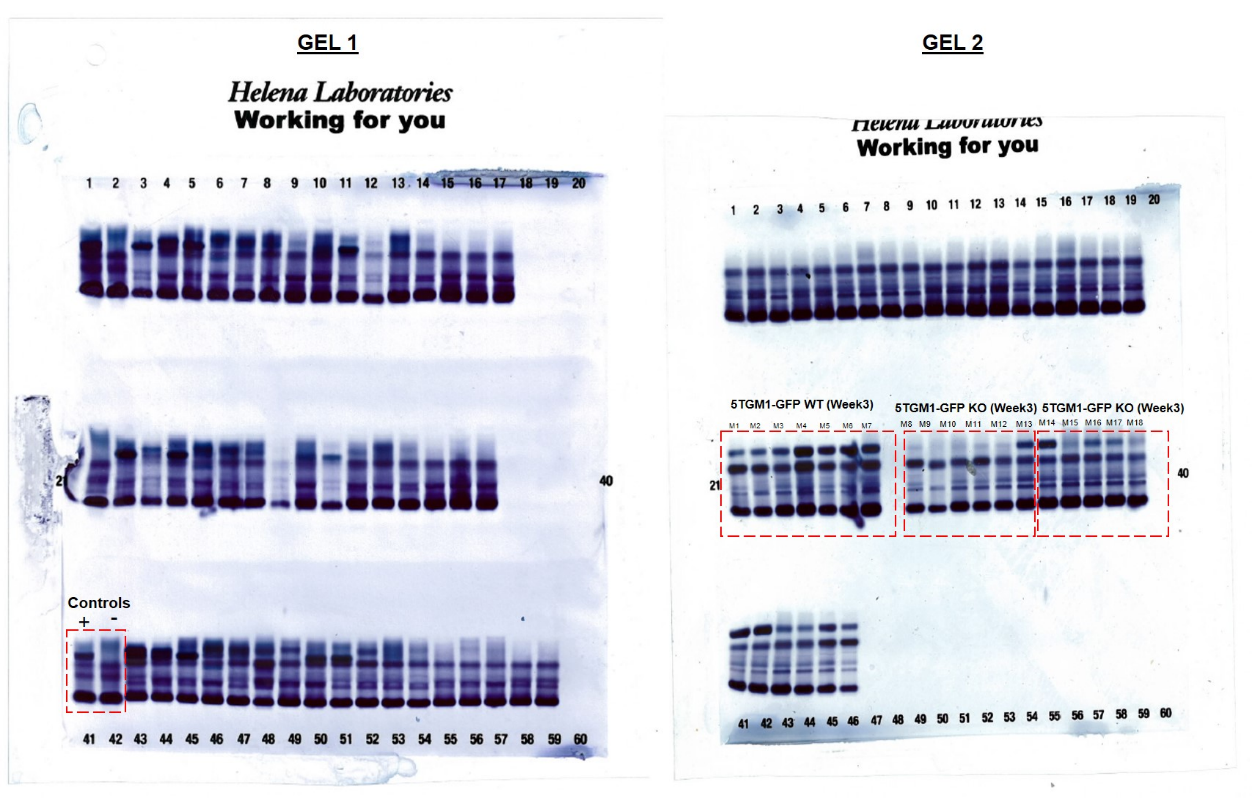


Here, we have included the full length gel pictures. We ran two gels with samples for different ongoing studies. The relevant samples for this manuscript are outlined in red. The gels were not incubated with any antibodies as this was serum protein electrophoresis experiment where electrophoresis separates proteins based on their physical properties, and the subsets of these proteins are used in interpreting the results.

We showed only the relevant part of the gels for this manuscript as the remaining samples on the gel are from different studies.
