# Supplementary material for: Ablation of VLA4 in multiple myeloma cells redirects tumor spread and prolongs survival
Source: Sci Rep. 2022 Jan 7;12:30. doi: 10.1038/s41598-021-03748-0 (PMC8741970; doi:10.1038/s41598-021-03748-0)
Supplement: Supplementary file 2 — Supplementary Information 2. [file 41598_2021_3748_MOESM2_ESM.docx]

**Ablation of VLA4 in multiple myeloma cells redirects tumor spread and prolongs survival**

Deep Hathi^1+^, Chantiya Chanswangphuwana^2,3+^, Nicholas Cho^1+^, Francesca Fontana^4+^, Dolonchampa Maji^1^, Julie Ritchey^2^, Julie O’Neal^2^, Anchal Ghai^5^, Kathleen Duncan^5^, Walter J. Akers^6^, Mark Fiala^2^, Ravi Vij^2^, John F. DiPersio^2^, Michael Rettig^2^, Monica Shokeen^1,5^*

^1^Department of Biomedical Engineering, Washington University in St. Louis, St. Louis, MO, USA

^2^Department of Medicine, Division of Molecular Oncology, Washington University School of Medicine, St. Louis, MO, USA

^3^Department of Medicine, Division of Hematology, Chulalongkorn University and King Chulalongkorn Memorial Hospital, Bangkok, Thailand

^4^Department of Medicine, Division of Cardiology, Washington University School of Medicine, St. Louis, MO, USA

^5^Department of Radiology, Washington University School of Medicine, St. Louis, MO, USA

^6^St. Jude Children’s Research Hospital, Memphis, TN, USA

^*^Corresponding author: [mshokeen@wustl.edu](mailto:mshokeen@wustl.edu)

^+^These authors contributed equally to this work

**Supplemental Figures.**

**
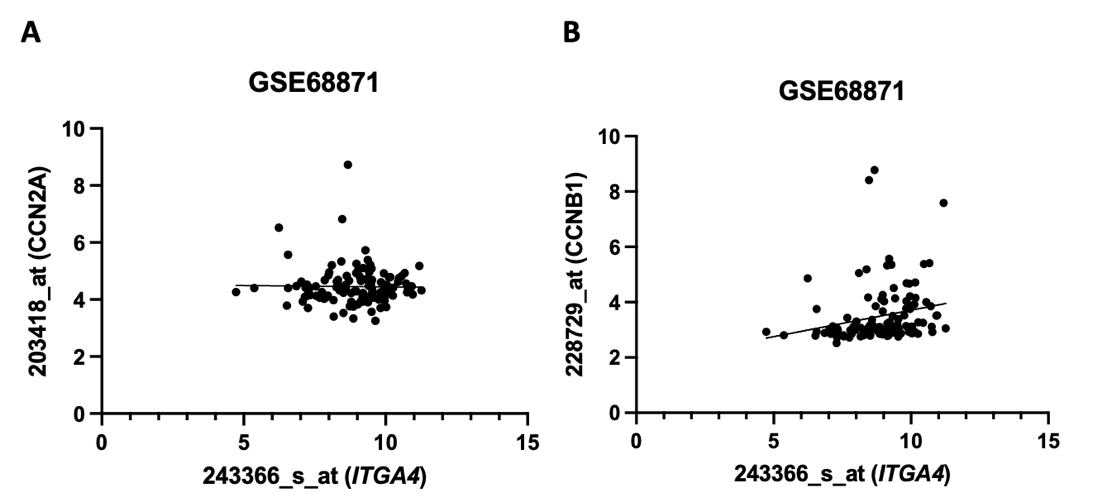
**

**Supplementary Figure 1.** Correlation of ITGA4 with **(A)** cyclin 2A and **(B)** cyclin 2B in the GSE68871 dataset. No correlation was found between the expression of ITGA4 and this proliferation-associated transcript. Correlation between ITGA4 and CCNB1 showed a P<0.05; however, a very low coefficient of determination (R=0.015) points to dubious significance of the association.


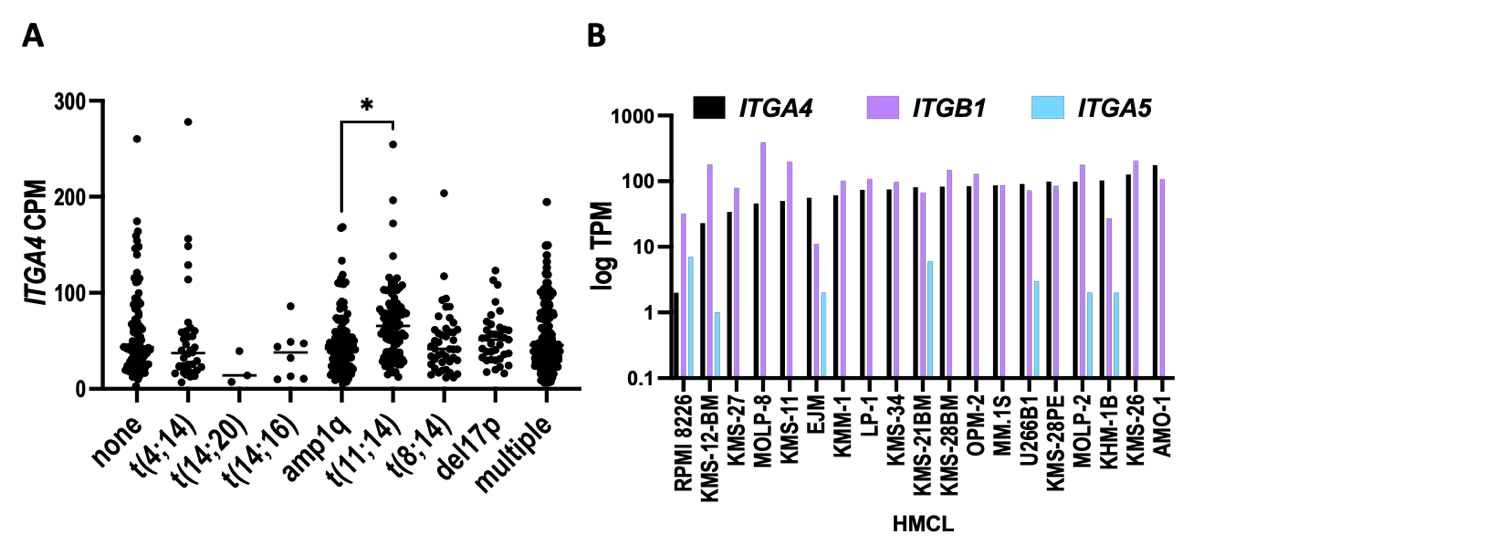


**Supplementary Figure 2.** **(A)** Expression of ITGA4 in patients from the CoMMpass dataset with no known cytogenetic abnormalities (none, N=97), two or more (multiple, N=234), or single cytogenetic abnormalities at t(4;14) (N=36), t(14;20) (N=3); t(14;16) (N=8),  t (11;14) (N= 91),   t(8;14) (N=44), amp1q (N=106), del17p  (N= 37). **(B)** Expression of *ITGA4*, *ITGB1*, and *ITGA5* in human myeloma cell lines. Arrow: KMS12 (t11;14)+





**Supplementary Figure 3.** The *in vivo* characteristics of the *Itga4* KO MM.1S-GFP cells were compared with the WT MM.1S-GFP cells in the intravenous (i.v) NSG mouse model. (**a**) The *Itga4* KO MM1.S-GFP cells were successfully generated. (**b**) There was the survival advantage in *Itga4* KO MM1.S-GFP engrafted mice. (**c**) The distribution of MM.1S cells in different tissues was presented with the percentage of MM.1S cells at the time of death. **(d)** Graph showing the number of mice that manifested with extramedullary plasmacytomas.

**Supplementary Figure 4. LLP2A-Cy5 uptake in VLA-4 expressing 5TGM1-GFP tumors *in vivo*.** **(a)** *In vivo* imaging of LLP2A-Cy5 fluorescence showed strong contrast and colocalization with 5TGM1-GFP tumors in C57Bl/KaLwRij mice bearing intramedullary 5TGM1-GFP myeloma tumors (3 weeks post-tumor inoculation (i.v.)). *In vivo* 3D fluorescence imaging was performed on IVIS Spectrum CT (PerkinElmer, Waltham, MA) with auto-exposure settings in the GFP wavelength (Ex./Em. 480nm/520nm) filter pair. 100µL of 25µM LLP2A-Cy5 was administered *via* lateral tail vein in 5TGM1-GFP WT (n = 2) and no tumor C57Bl/6 KaLwRijmice (female, n = 2) 3 weeks post tumor cell implantation. The images were acquired 18 h post administration of the LLP2A-Cy5 contrast agent. **(b)** *Ex vivo* biodistribution of LLP2A-Cy5 and GFP fluorescence 18hr post LLP2A-Cy5 i.v. injection showed significant uptake of LLP2A-Cy5 in tumor tissue (***p* < 0.01, ****p* < 0.001 1-way ANOVA with Bonferroni multiple comparison tests of LLP2A-Cy5 normalized fluorescence relative to muscle; ^†††^*p* < 0.001, ^††^*p* < 0.01 1-way ANOVA with Bonferroni multiple comparison tests of LLP2A-Cy5 normalized fluorescence in tumor-bearing mice relative to LLP2A-Cy5 normalized fluorescence in non-tumor bearing mice (n=3)). **(c)** Strong LLP2A-Cy5 uptake in VLA4^+^ 5TGM1-GFP tumor cells in the BM was observed. GFP^+^ cells were co-stained with PE-conjugated anti-mouse CD49d and eFluor450-conjugated anti-mouse CD29 antibodies (middle; Thermo Fisher Scientific) to identify VLA4^+^ cells. Specificity of LLP2A-Cy5 to VLA4^+^ 5TGM1-GFP tumor cells was assessed (right). **(d)** Strong LLP2A-Cy5 signal was also seen in VLA4^+^ 5TGM1-GFP tumor cells in the spleen, with GFP^+^ cells (left) gated with VLA4 expression (middle). LLP2A-Cy5 binding to VLA4^+^ GFP^+^ was measured (right).

**Supplementary Figure 5.** Representative 2D GFP and Cy5 *ex vivo* images of excised bone and non-tumor tissue from WT and KO mice. Representative images of 1) blood 2) heart 3) lung 4) liver 5) spleen 6) kidney 7) leg bones 8) muscle 9) small intestine


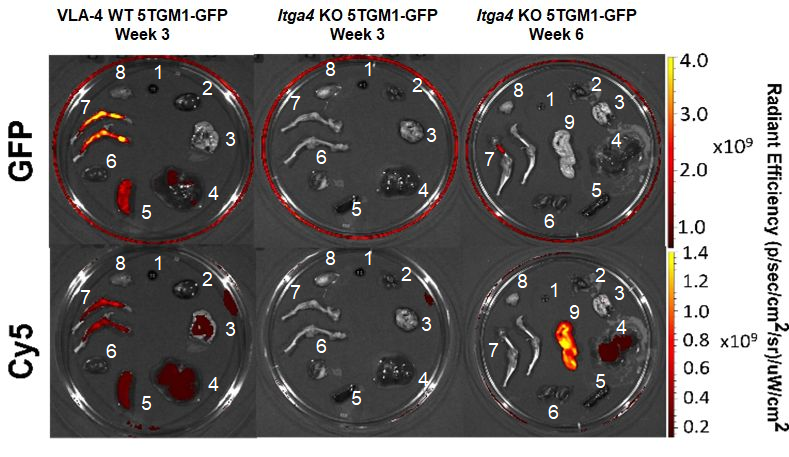


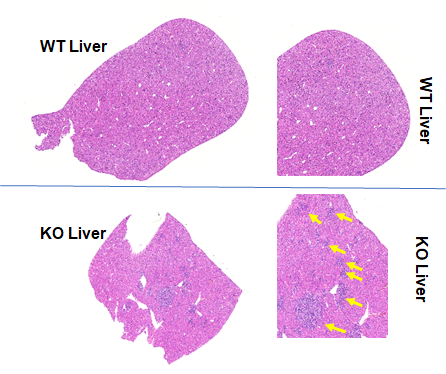


**Supplementary Figure 6.** Representative histology slides of liver at week 3 post-tumor inoculation in WT and KO mice. In the high magnification slice on right, yellow arrows are pointing to the tumor cells in the KO tumor mice. Tumor cells are not visible in the liver of WT mice at week three.


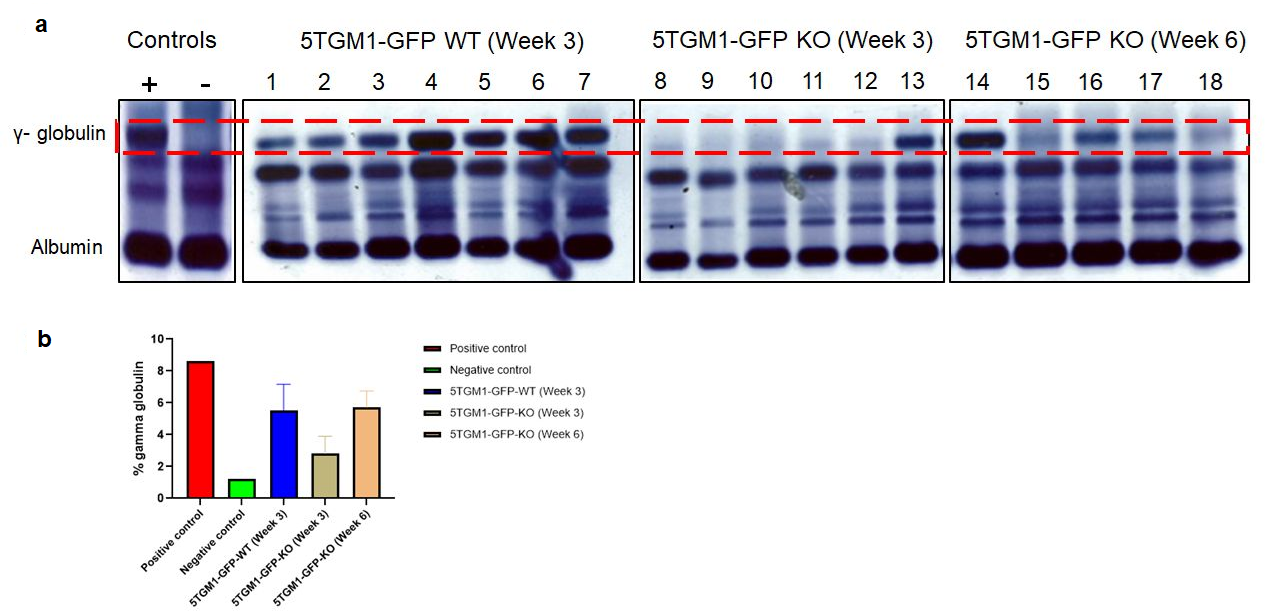


**Supplementary Figure 7.** **(a)** Gel picture of serum protein electrophoresis (SPEP) for the mice, **(b)** % Gamma globulin fractions in total serum protein. (+: positive control, -; negative control). *Additional information about the gel is below:


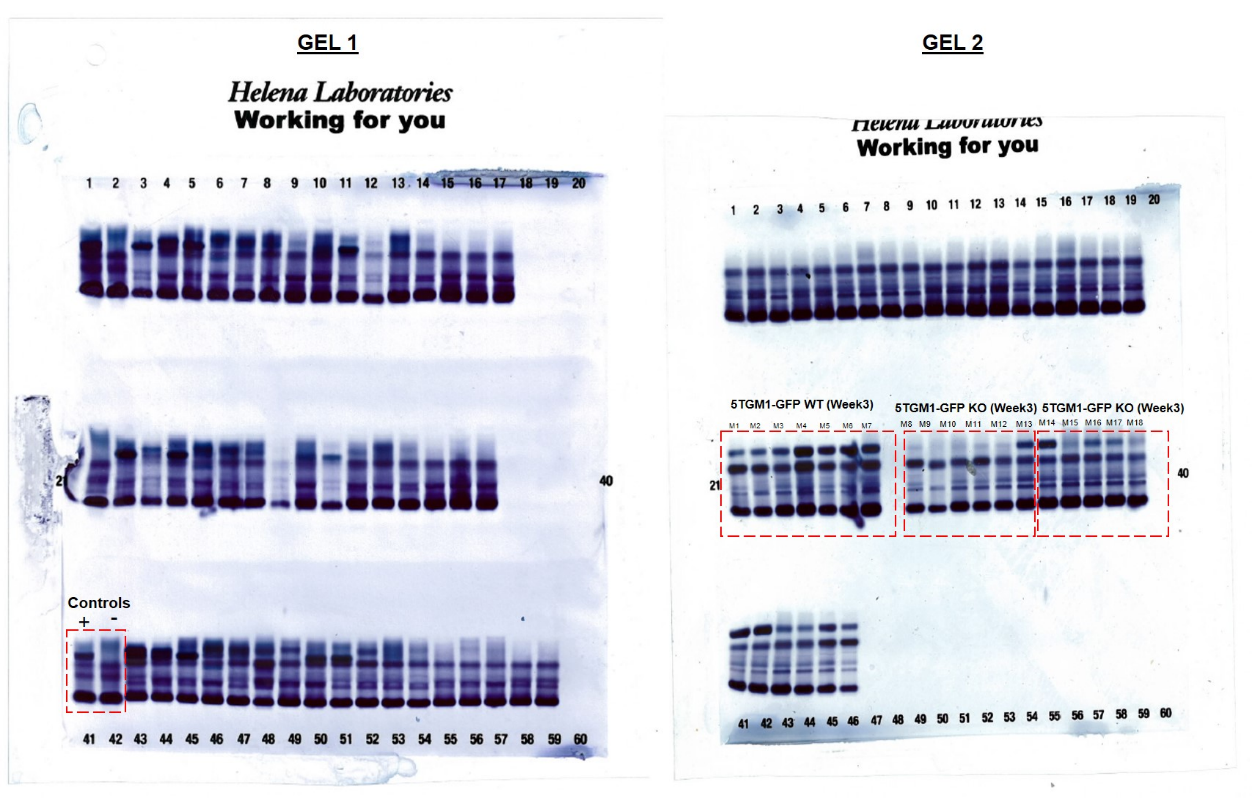


Included here are the full length gel pictures. Two gels were ran with samples for different ongoing studies. The relevant samples for this manuscript are outlined in red. The gels were not incubated with any antibodies as this was serum protein electrophoresis experiment where electrophoresis separates proteins based on their physical properties, and the subsets of these proteins are used in interpreting the results.

We showed only the relevant part of the gels for this manuscript as the remaining samples on the gel are for different studies.
